# Exploring consumer acceptability of leafy greens in earth and space immersive environments using biometrics

**DOI:** 10.1038/s41538-024-00314-6

**Published:** 2024-10-09

**Authors:** Claudia Gonzalez Viejo, Natalie Harris, Eden Tongson, Sigfredo Fuentes

**Affiliations:** 1 Digital Agriculture, Food and Wine Research Group. Faculty of Science, The University of Melbourne, VIC, 3010 Australia; 2 Centre of Excellence in Plants for Space. Australian Research Council, University of Adelaide (Lead University), Glen Osmond Rd, Adelaide, SA Australia; 3 Tecnologico de Monterrey, School of Engineering and Science, Ave. Eugenio Garza Sada 2501, Monterrey, NL 64849 México

**Keywords:** Emotion, Sensory processing

## Abstract

Novel research on food perception is required for long-term space exploration. There is limited research on food/beverage sensory analysis in space and space-simulated conditions, with many studies presenting biases in sensory and statistical methods. This study used univariate and multivariate analysis on data from pick-and-eat leafy greens to assess self-reported and biometric consumer sensory analysis in simulated microgravity using reclining chairs and space-immersive environments. According to ANOVA (*p* < 0.05), there were significant differences between interaction room × position for head movements; besides, there were non-significant differences in the interaction samples × environment. On the other hand, there were significant differences in the sample×position interaction for all liking attributes. Results from multivariate analysis showed effects on self-reported, physiological, and emotional responses of samples in space-related positions and environments related to sensory perception changes. Non-invasive biometrics could offer a powerful tool for developing digital twins to assess genetically modified plants and plant-based food/beverages for long-term space exploration.

## Introduction

NASA has scheduled extended space voyages, intending to send crews to the Moon via the Artemis program in 2030 and Mars by 2040. These missions will require the development of specialized plants, most probably genetically modified (GM), to provide not only fresh food in the potential form of fruits, vegetables, and leafy greens but also food micro and macro-nutrients such as minerals, vitamins, proteins, carbohydrates, and fat for complete nutritious foods and beverages as well as sensory attributes such as texture and flavor for astronaut’s sustenance^1^.

The latter is one of the objectives of a newly funded Centre of Excellence in Plants for Space (CoE-P4S), which started in January 2024 until 2030 by the Australian Research Council, involving five Australian Universities and 38 national and international partners, including Axiom and NASA^2^. Some early candidate plants have been identified by the CoE-P4S, such as Duckweed (*Lemna minor L*.), Tomato (*Lycopersicum sculentum*), and Arabidopsis (as a rapid model plant), among others^1^.

These types of research aiming to produce GM plants and food materials derived from the same for long-term space explorations are confronted with two main constraints from the sensory evaluation perspective: (i) on-Earth experimentation requiring the closest simulation to microgravity and space environment effects of participants and (ii) problems conducting sensory sessions of GM plants and plant-based food and beverage products, which will require extensive human ethic applications and processes.

Considering these constraints, there has been limited research assessing the changes in sensory perception on Earth using simulated microgravity positions through reclining hospital beds compared to space microgravity conditions^3–7^. Early results from these studies found changes in aroma perception from participants, which were reduced, and taste perception enhanced. A recent study has reported reduced sensitivity and perceived intensity of aromas when in a supine (reclined) position simulating microgravity, which is in accordance with previous research^6–10^. On the other hand, some authors have reported increased perceived intensity of basic tastes in the supine position^8,10,11^ and space conditions for sweet and salty tastes^12^.

Concerning the validity of these studies^4–8^, some previous research has common problems in sensory science, which have led to contradictory findings when simulating microgravity on Earth for sensory trials. Some of these problems relate to using very few (lower than six) untrained panelists, which could not be representative. A more recent publication described a study using 12–14 well-trained panelists to assess specific changes in aroma, taste, and mouthfeel (trigeminal sensations) perception using simulated microgravity positions through reclining chairs^10^. The main results from this study coincided with previous publications reporting changes perceived by astronauts in space^12^, showing a decrease in aroma perception and an increase in basic tastes in the simulated microgravity seating position. A new finding from that study was that there was a perceived increase, although not significantly different, in mouthfeel, which had not been studied in space or space-simulated conditions beforehand. Furthermore, space environments must be introduced to assess consumer perception changes since they may play an important role in the sensory perception of food and beverages.

It has been shown in previous studies that the environment plays an important and sometimes critical role in the sensory analysis of participants^13^. For example, Torrico et al.^14^ tested consumers' perception of wine in different real (immersive) and virtual reality (VR) environments and found differences in aroma perception with enhanced intensities in both immersive and VR environments compared to traditional sensory booths. Besides, Gouton et al.^15^ found better performance when conducting sensory evaluation of sandwiches in immersive and VR settings than traditional sensory booths. Delarue et al.^16^ tested consumers acceptability of alcohol-free beer in traditional sensory booths and immersive environments and found that the latter can better uncover situational appropriateness. Hence, this research proposed the use of immersive space environments for sensory perception studies on consumers.

In terms of using GM plants and plant materials in the future, researchers have proposed an alternative to bypass the use of humans for sensory analysis of fresh plant materials and products developed from the same plants using Artificial Intelligence (AI), specifically through the development of Digital Twins (DT) for sensory analysis of GM plants and food/beverage products derived from them^10,17^. The first step to constructing these DTs, as preliminary work before GM plants and food are produced for long-term space exploration, is to test novel digital sensory technologies incorporating non-destructive biometrics to assess physiological changes and emotional responses of participants in immersive space sensory sessions^18^.

Considering the latter approaches, pick-and-eat leafy greens were chosen as assessment products, which could be considered the simplest systems to develop DTs from plant production, harvest, and sensory perception in immersive space and microgravity-simulated conditions. Hence, this research aimed as a first approach to assess consumers’ biometrics and acceptability of pick-and-eat leafy greens produced in small-scale robotic farming systems in space-simulated conditions. The latter would produce enough digital information from consumers related to their self-reported data plus physiological and emotional responses for future development of DT using GM plants and plant products.

## Results and discussion

### Analysis of variance (ANOVA)

**Fig. 1:**
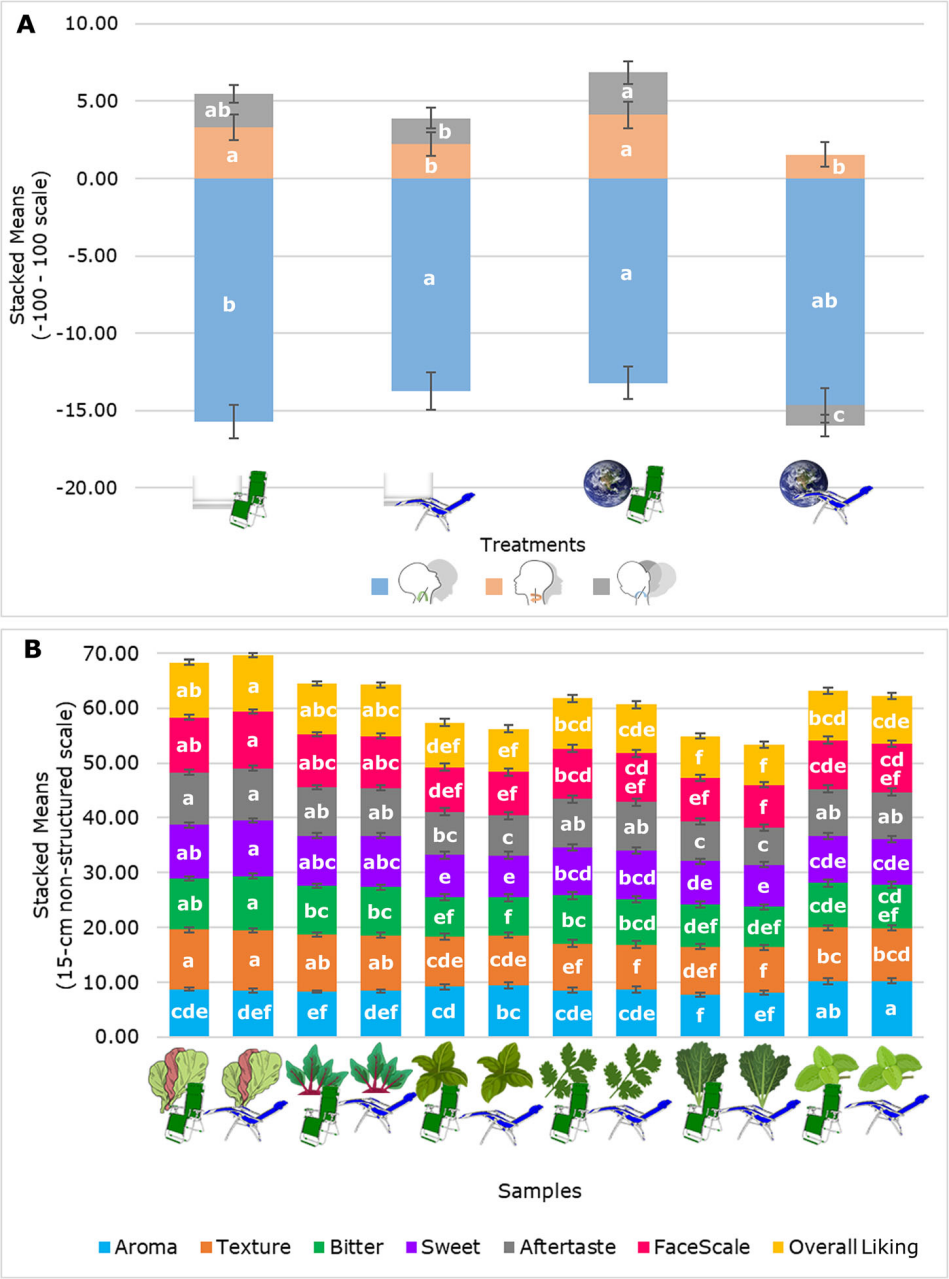
Figures showing mean values and ANOVA results. Stacked bar graphs showing the variables with significant differences (*p* < 0.05) in (**A**) seating position*environment interaction and **B** sample*seating position interaction, according to the ANOVA and Fisher’s least significant difference test (LSD; α = 0.05), depicted by different letters (a–f). Error bars were calculated based on standard error (*n* = 51). Variables in (**A**): Jaw , Pitch , and Roll  head movements.

There were no significant differences (*p* *>* 0.05) in the self-reported acceptance responses considering the interaction of environment and seating position. However, Fig. 1A shows significant differences (*p* *<* 0.05) in the head movements from the interaction of environment and seating position. It can be observed that in the neutral environment, the pitch  head position was lower (downward movement) in the normal position  than in the microgravity position  and the normal position in the space environment . This means that in the neutral environment with microgravity position , consumers moved their head upwards in a possible attempt to rest their heads on the chair to compensate for gravity in that position and avoid an awkward position of the neck and head downwards. A similar response was shown for the normal position in the space environment , possibly due to participants looking (directly or indirectly) at the space environment displayed on the 180° screen.

Besides, in the microgravity position , both environments presented similar head movements for yaw ; even though all treatments had positive values (rotation to the left) for this variable, those using microgravity position  in both environments had less movement to the sides than the normal position . This may be due to the microgravity position being more relaxing and providing a weightless sensation^19^ and, therefore, reducing the need to move the head sideways. On the other hand, the roll  head movement for the microgravity position  in space environment  had negative values (tilting to the right), while other treatments presented positive values (tilting to the left). This coincides with findings from a recent study in which tilting the head to the right was found in the microgravity position  and was attributed to enhanced taste intensity perception^10^; furthermore, in other studies, tilting the head to the left has been associated with social anxiety and increased self-conscious emotions^20^. Tables S1 and S2 in supplementary material show the mean values, standard error, and *p* value from ANVOA of all self-reported and biometrics of the environment × seating position interaction.

On the contrary, the interaction of samples and environment did not present significant statistical differences for any sensory self-reported or biometric variable; mean values, standard error and *p* value from ANVOA of all biometrics and self-reported responses of the samples × environment interaction are shown in Tables S3 and S4. Besides, Fig. 1B shows that all self-reported responses had significant differences (*p* *<* 0.05) in the interaction of samples and seating position. Since the same samples in both positions shared at least one letter of significance, it means that the major differences were due to the different samples but not to the seating position itself. In general, it can be observed that sweet basil  was the most liked in aroma, while lettuce  was the most liked for texture, bitterness, sweetness, aftertaste, and overall liking, as well as having the highest values for FaceScale (positive emotion). On the other hand, kale  was the least liked for aroma, overall liking, and most negative emotion (lowest in FaceScale), and one of the least liked for texture, sweetness, and aftertaste. Thai basil  had the lowest liking for bitterness and shared the lowest liking for sweetness and aftertaste. Coriander  was one of the least liked in texture and, along with beetroot , shared similarities with other samples for all other descriptors. Tables S5 and S6 in supplementary material show the mean values, standard error and *p* value from ANVOA of all self-reported biometrics of the samples × seating position interaction.

Results from the ANOVAs in this study showed that in complex research involving multiple samples and treatments, in this case, environments and seating positions, there is a need to conduct multivariate data analysis since this provides more findings and insights into the possible causes of those findings than a simpler univariate analysis (i.e., ANOVA). This may be considered as another limitation of most of the existing studies related to food sensory perception in simulated microgravity conditions as these often report ANOVA or t-test to assess significant differences^4–8,11,21^.

### Multivariate data analysis

#### Correspondence analysis

Previous to the CA, Cochran Q test was conducted for the interaction of sample × room × position; it was found that there were significant differences (*p* < 0.05) between sample × room × position for , , , , , , , , and ; however, from the pairwise comparison test, differences were only found for , , , and  (supplementary material Table S7). Based on the average percentage of selection the three most selected emojis were  (average = 26%; max selection in at least one sample = 37%),  (average = 24%; max = 37%),  (average = 23%; 45%), and the three least selected were  (average = 4%; max = 10%),  (average = 3%; max = 8%),  (average = 1%; max = 4%).

**Fig. 2:**
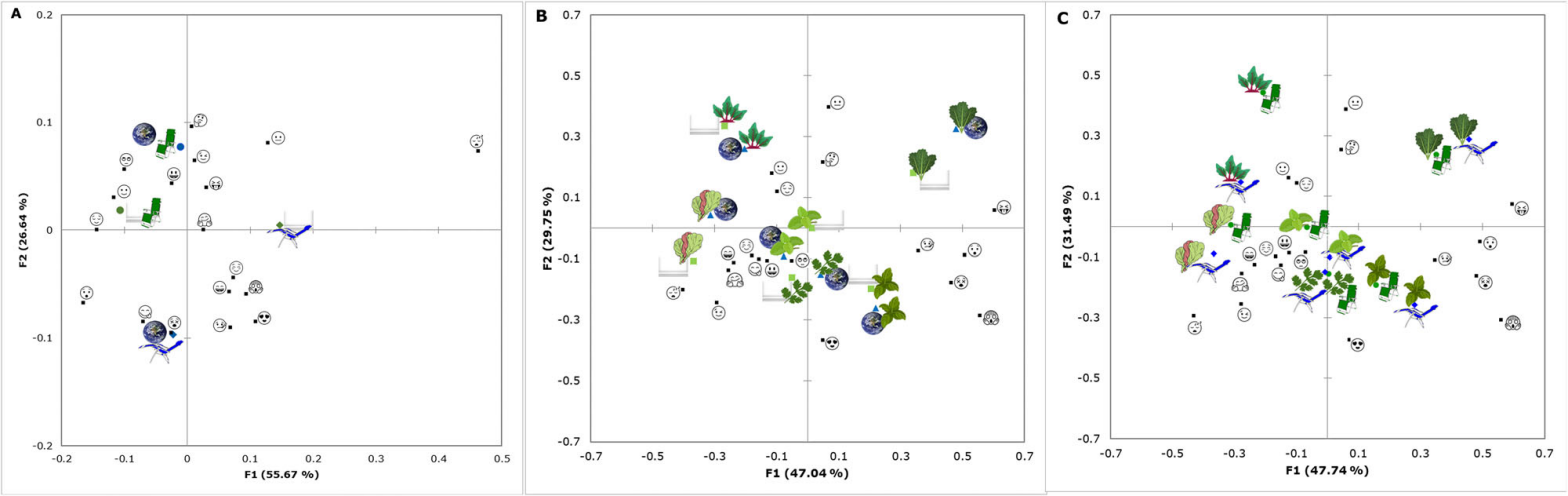
Correspondence Analysis of the Check all that Apply of Emojis. Correspondence analysis of the check all that apply test using emojis for the mean values of (**A**) seating position and environment, **B** sample and environment, and **C** sample and seating position. F1 and F2: factors one and two. Images represent Thai basil , sweet basil , coriander , kale , lettuce , beetroot , Space  environment , neutral environment , normal position, and microgravity position .

Figure 2 shows the CA of the (i) environment and seating position (Fig. 2A), (ii) sample and environment (Fig. 2B), and (iii) sample and seating position (Fig. 2C) interaction for the CATA of emojis. In the (i) environment and seating position CA, factor one (F1) represented 55.67% of data variability, while factor two (F2) accounted for 26.64%, with a total of 82.31%. In the (ii) sample and environment CA, factors represented a total of 76.79% of data variability (F1: 47.04%; F2: 29.75%). Besides, factors one and two represented 79.23% (F1: 47.74%; F2: 31.49%) of data variability for the (iii) sample and seating position CA.

As shown in Fig. 2A, the normal seating position  in both environments was associated with positive self-reported emotions through emoticons, such as , , , , and , as well as some neutral emotions, such as  and . On the other hand, the microgravity position in the space environment  was related to mixed emotions such as , , , and . Besides, the microgravity position in the neutral environment  was associated with , , , and . The mixed emotions elicited by the microgravity position in both environments may be due to the unexpected and unfamiliar position, as this is not the normal setting for food consumption. The emotions on the microgravity-simulated position in the space environment  may be due to the unfamiliar yet relaxing and pleasant settings, as verbally described by participants at the end of the session. Even though many publications tested sensory acceptability in different environments, either using physical rooms or virtual reality not only for space but for different Earth settings^13–15,22,23^, and a few publications testing sensory perception in normal and reclining position simulating microgravity^3–5^, none of those have assessed the emotional responses to the interaction of both environment and seating position nor physiological responses, which are presented later.

Figure 2B shows no effect of the environment on the emotions elicited by the samples since the same sample in both environments was grouped for all individual leafy greens. Beetroot  in both environments was associated with emotions such as , and , sharing these associations with the lettuce  in space environment . Furthermore,  lettuce was grouped with sweet basil  and coriander , all in both environments. These results were related mainly to positive emotions such as , , , , and , as well as other emotions such as  and . The association of samples such as , and  with positive emotions may be due to their characteristic sweeter and neutral taste compared to other leafy greens, the higher familiarity of consumers with lettuce, and the specific quality trait of crunchiness^24–26^. Besides, Thai basil  in both environments was associated with more negative emotions such as , , , and , and shared these associations with coriander  in space environment . This may be due to the spicy or pungent flavor in Thai basil, which may be overwhelming, and the soapy flavor that some consumers may perceive in coriander due to their genetics^27,28^. Kale  in both environments was separated from all other samples and mainly related to emotions such as , , and  and negatively related to all positive emotions. This may be due to the bitter taste characteristic of kale, which tends to be rejected by consumers as a genetic and neurological response from a human evolutionary trait related to its association with poison avoidance^29–31^.

Figure 2C shows no major effect of the seating position on the emotions elicited by the samples. Associations and groupings in these CA were similar to those in the samples/environment CA. Beetroot  in both seating positions was mainly associated with  and , and shared these emotions with lettuce  and sweet basil  in normal position . Lettuce  was grouped with coriander  and sweet basil in both seating positions, and they were related to emotions such as , , , , , and . Furthermore, Thai basil  was associated with , , , , and . On the other hand, kale  was related to , , and . Despite the samples in both positions being grouped, a discernible pattern can be observed since the samples tasted in the normal position  were closer to the origin (center) of the PCA, whereas those tasted in the microgravity position  were positioned farther. This suggests that emotions experienced in the latter position might be more intense, possibly attributed to the relaxing effect of the weightless sensation induced by the microgravity environment^19^.

#### Principal components analysis

**Fig. 3:**
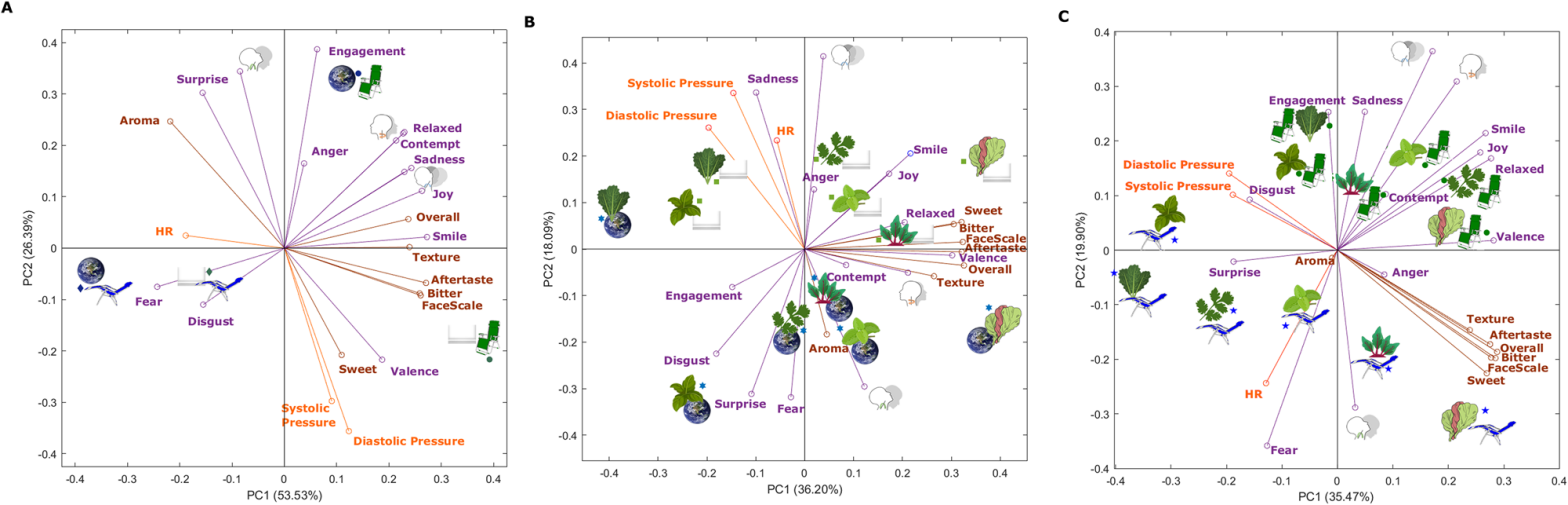
Principal Components Analysis. Principal components analysis of the self-reported liking (brown), emotional (purple), and physiological responses (orange) for the mean values of (**A**) seating position and environment, **B** sample and environment, and **C** sample and seating position. PC1 and PC2: principal components one and two. Images represent Thai basil , sweet basil , coriander , kale , lettuce , beetroot , Space environment , neutral environment , normal seating position , and microgravity position . Other abbreviations are shown in Tables 1, 2.

Figure 3 shows the PCAs for the mean values of the interactions between the (i) seating position and environment (Fig. 3A), (ii) sample and environment (Fig. 3B), and (iii) sample and seating position (Fig. 3C). In the (i) seating position and environment, principal component one (PC1) represented 53.53% of data variability, while principal component two (PC2) accounts for 26.39%, with a total of 79.92%. For the (ii) sample and environment PCA, PC1 represented 36.20%, and PC2 18.09%, accounting for a total of 54.29%. On the other hand, for the (iii) sample and seating position, the PCs represented a total of 55.37% (PC1: 35.47%; PC2: 19.90%) of data variability. The cutoff point of 60% of data variability explained by the total of both PC1 and PC2 was considered to test significance^32^; however, this was deemed for PCA in Fig. 3A, which had 79.9%, and it was decided to keep the same variables for the other two PCAs (Fig. 3B, C) that were close enough to 60% for better comparison and representation between the three PCAs.

Figure 3A shows that, according to the factor loadings (FL), PC1 was mainly represented by aftertaste (FL = 0.27), smile (FL = 0.27), bitter (FL = 0.26), aftertaste (FL = 0.26), and joy (FL = 0.26) on the positive side, and by fear (FL = −0.24) and aroma (FL = −0.22) on the negative side of the x-axis. Besides, PC2 was characterized on the positive side of the y-axis by engagement (FL = 0.39) and pitch  (FL = 0.34) and on the negative side by diastolic (FL = −0.36) and systolic pressure (FL = −0.30). It can be observed that the microgravity position in both  environments was grouped and associated with fear, disgust, and HR, which can be explained by this position being new to consumers and not the usual eating setting. The microgravity position in both environments is also positively associated with aroma liking. This may be due to the reduced aroma perception reported in previous studies due to the microgravity position^4,10^. Therefore, a milder, less overwhelming aroma may contribute to higher acceptability. The normal position  in neutral environment  was associated with FaceScale, valence, and liking of bitterness, sweetness, aftertaste, and texture, which might be attributed to the usual environmental settings when eating. In contrast, the normal position  in the space environment  was mainly related to engagement, anger, relaxed, contempt, and yaw .

Figure 3B shows that PC1 was characterized by overall liking (FL = 0.33), sweet (FL = 0.32), aftertaste (FL = 0.32), and FaceScale (FL = 0.32) on the positive side of the axis, and by diastolic pressure (FL = −0.20) and disgust (FL = −0.18) on the negative side. On the other hand, PC2 was mainly represented on the positive side of the axis by roll  (FL = 0.41), systolic pressure (FL = 0.34), and sadness (FL = 0.34), and on the negative side by fear (FL = −0.32) and surprise (FL = −0.31). Even though there were no significant differences found in the ANOVA, in the PCA, it can be observed that there is a clear separation of both environments along the PC2 with samples tasted in the neutral environment  located on the positive side of PC2 and all samples in the space environment , except for kale , on the negative side of PC2. Coriander , lettuce , sweet basil , and beetroot  samples tasted in the neutral environment  were grouped and associated with descriptors such as roll , smile, joy, relaxed, sweet and bitter liking, and FaceScale. While kale  in both environments and Thai basil  in neutral environment  were related to HR, systolic and diastolic pressure, and sadness. This may be due to the higher bitterness in these samples that elicit an alertness state in the brain and physiological responses due to the association with poisonous substances^29–31^. On the other hand, Thai basil  and coriander  in the space environment  were associated with engagement, disgust, surprise, and fear, while beetroot , sweet basil  and lettuce  in the space environment  were mainly related to the aroma, pitch , texture, and overall liking. The possible explanation of the samples in the space environment  being positively related to aroma liking may be due to the reduction in aroma intensity perception, as explained in the previous paragraph of this paper^4,10^.

In Fig. 3C, it can be observed that PC1 was represented on the positive side of the axis by overall liking (FL = 0.29), FaceScale (FL = 0.28), and valence (FL = 0.28), and on the negative side by diastolic (FL = −0.20) and systolic pressure (FL = −0.19), and surprise (FL = −0.19). Principal component two was characterized by roll  (FL = 0.36) and yaw  (FL = 0.31) on the positive side and by fear (FL = −0.36) and pitch  (FL = −0.29) on the negative side of the axis. Samples tasted in both seating positions were separated in the PC2 with those in the normal position  located in the positive side of PC2 along with Thai basil  in the microgravity position , and the rest of the samples in the microgravity position  were located in the negative side of PC2. All six samples in normal position  were grouped and associated with descriptors such as engagement, sadness, roll , yaw , smile, relaxed, and valence. Thai basil , kale , coriander , and sweet basil  in microgravity position  were grouped and related to surprise, systolic and diastolic pressure, HR, disgust, and aroma. While beetroot  and lettuce  in microgravity position  formed another group and were associated with liking of all descriptors except for aroma and pitch . This is in accordance with the results of the CA of emojis in which beetroot and lettuce were associated with the most positive emotions; as explained before, this may be due to their sweet, less bitter taste. The association of beetroot  and lettuce  samples tested in microgravity position  with liking of sensory descriptors may be attributed to the findings reported in previous studies with taste perception being intensified^10,12^, which may have contributed to higher sweetness in these two samples.

The multivariate data analyses showed that the differences between all tested conditions may be useful to develop machine learning models to model and find further patterns among the biometric data to target self-reported sensory responses to construct digital twins further to simulate GM plant foods and their acceptability for consumption in space.

Multivariate data analysis is also commonly used as parameter engineering for supervised machine learning and AI modeling strategies^33,34^. The latter allows for increasing accuracy of models based on patterns found within the data and for the machine learning model to generate patterns related to the weights and biases found within the data.

Several machine learning models have been developed using digital information measured from plants, food products, and beverages to target specific sensory perceptions, such as liking, aroma profile, and taste. Some research done using this approach involves the assessment of wine quality and sensory descriptors based on berry cell dead and canopy architecture^35^, prediction of wine sensory profile using weather and water management data^36^, and prediction of aromas using canopy architecture of cocoa trees as inputs^37^.

A limitation of the study is the lack of access to test with astronauts since there is only one astronaut in training in Australia and the lack of feasibility to conduct tests in real Space environment. However, results from this study show that using immersive environments including the simulated microgravity position provide a good approach to simulate Space conditions on Earth, this may be evidenced by the separation of samples tested in both environments in multivariate data analysis. Besides, the findings of this study underscore the significance of employing multivariate data analysis in complex research scenarios, particularly in novel investigations involving multiple samples and treatments within varying space environments and seating positions to simulate microgravity changes in sensory perception. It has been shown that multivariate analysis offers a more comprehensive understanding of potential causal factors compared to simpler univariate methods. This multivariate data analysis information proves instrumental in developing machine learning models, leveraging biometric data to construct digital twins for simulating genetically modified plant foods. These models have the potential to enhance our understanding of self-reported sensory responses and physiological and emotional responses obtained objectively using digital technologies to assess the acceptability of such foods for consumption in space.

The broader scientific context highlights the development of machine learning models using digital information from plants, food products, and beverages to target specific sensory perceptions. These models aim to predict preferences related to liking, aroma profiles, and taste, emphasizing the growing role of advanced analytical techniques in advancing our understanding of complex sensory phenomena for long-term space exploration.

## Materials and methods

### Leafy green samples description

**Fig. 4:**
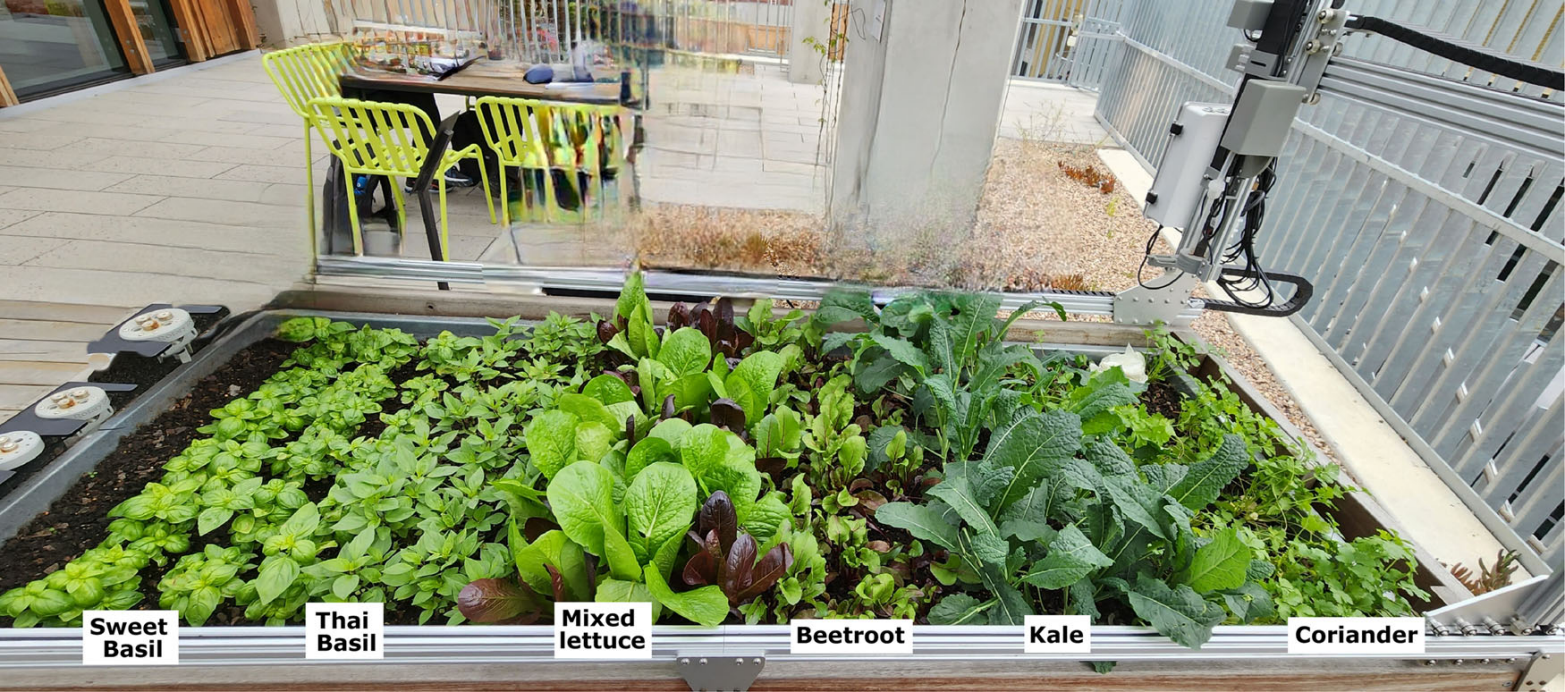
Farmbots with Samples before Harvest. Image depicting one of three Farmbots planted with six different leafy greens used for the consumer's sensory study, namely Sweet Basil, Thai Basil, Mixed Cos Lettuce, Beetroot, Kale, and Coriander. Photo captured by the authors.

Six leafy greens were cultivated in three FarmBots (FarmBot, San Luis Obispo, CA, USA) located at the Student Precinct at The University of Melbourne (UoM). These samples comprised Thai basil (*Ocimum tenuiflorum* ‘Krapao’) , sweet basil (*Ocimum basilicum*) , coriander (*Coriandrum sativum*) , kale (*Brassica oleracea* var. sabellica) , mixed cos lettuce (*Lactuca sativa L*. var. longifolia) , and beetroot (*Beta vulgaris*)  (Fig. 4). The selection of these samples was based on the little research regarding the sensory of foods in Space-simulated conditions considered for long-term missions, most is only for short-term, in which astronauts will be required to produce their own food, it is important to evaluate individual fresh ingredients to understand their acceptability and subconscious responses. Once this is understood, more studies can be conducted using salads or other dishes, including these as ingredients. In a previous study from Mauerer et al*.*^38^ a survey was conducted with former space crew members revealed that, for example, 73% of respondents missed consuming basil, 57% lettuce, and 5% coriander while in Space. Besides, 23% of respondents mentioned they preferred to pick-and-eat to consume fruits, leafy greens and herbs, and 64% preferred to pick/cut them and prepare them as salad.

On the previous day of the sensory session, samples were harvested and taken to the food-grade sensory laboratory located at UoM, Parkville Campus, Australia, to be properly washed. The best quality specimens for each sample were selected based on no visible defects or damage. Samples were stored in tightly closed containers with wet paper towels to retain moisture and kept in the refrigerator at 5 ^o^C for 24 h to preserve freshness.

### Sensory evaluation sessions and treatments

The sensory session was conducted in the immersive rooms in the Arts West building belonging to the Faculty of Arts at UoM. Leafy green consumers were recruited via email and newsletter announcements distributed among staff and students from UoM. A total of 51 consumers (37% males, 63% females; 18–51 years old) participated in the study. All participants were regular leafy greens consumers (at least once a week), either Australian or Asians living more than two years in Australia. The power analysis calculated considering overall liking mean values and standard deviation was conducted using SAS® v. 9.4 (SAS Institute, Cary, NC, USA), this stated that 51 consumers are statistically enough to find significant differences between samples (1 – β = 0.97), which meets the requirement of minimum 80% Power^39^. The number of participants is also within the range (adequate: 20–150; usual: 50–100) stated by Mammasse and Schlich^40^.The protocol used in this study was approved by the Human Ethics Committee from UoM, Australia (ID: 1953926.6), and participants were asked to sign a consent form before the trial.

**Fig. 5:**
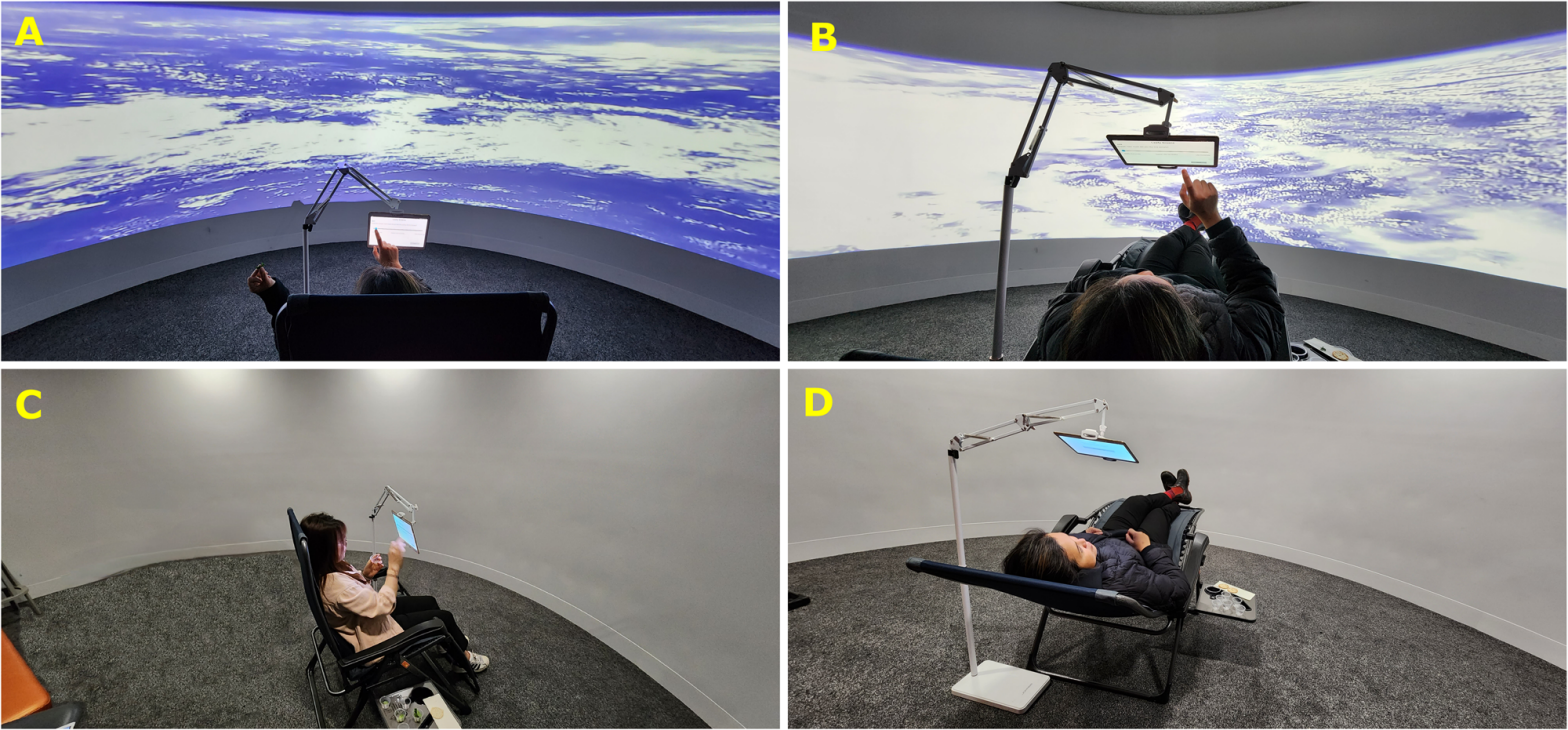
Immersive Rooms and Reclining Chairs used as Treatments. Images showing the different treatments used during the study, where (**A**) shows the Space environment with normal seating position, **B** the Space environment with simulated microgravity seating position, **C** the neutral environment with normal seating position, and **D** the neutral environment with simulated microgravity seating position. Figures **A** and **B** used a 180° screen projecting a video of Earth seen from Space. Figures **C** and **D** are from an adjacent second room with a neutral 180° screen without projections. Participants appearing in the images consented the publication of this Figure. Photos captured by the authors.

Two immersive rooms were used for the study. Immersive room one (Figs. 5A, 2B) displayed a video of the Earth seen from Space, while adjacent room two (Figs. 5C, 2D) had a white background simulating neutral conditions. The study consisted of four treatments within the two immersive environments (i) Earth video seen from Space projected on a 180° screen , and (ii) a white background 180° screen (neutral) . Two seating positions were implemented: (i) normal (90–270°)  and (ii) microgravity to simulate microgravity effects (0–170°)  as described by ref. ^10^. The latter position is not flat, allowing the participants to not look at the ceiling; hence, they are able to see the full screen directly and through peripheral sight. To achieve the different seating positions, Timber Ridge reclining chairs (Westfield Outdoors®, Indianapolis, IN, USA) were used; each of these had a tablet stand holding a 10” Android tablet.

All samples were labeled with three-digit random codes, which varied for each treatment. The entire set of samples was provided to participants at the start of the test, and they were instructed to select the sample that the questionnaire showed since these were tasted in random order. Water and crackers were provided as a palate cleanser between samples. Once participants ended a test in the first room/environment (either space immersive or neutral) and normal position, they were moved to the microgravity position, and once concluded, they were instructed to follow the same protocol in the other room/environment. All consumers participated in the four treatments, and the order of the rooms in which they started was randomized to reduce bias. The sessions were run with one participant per room at a time, and participants were allowed to take a 40–60-min break between treatments.

The questionnaire was displayed on Android tablets using the BioSensory© App (The University of Melbourne, Parkville, VIC, Australia)^18^. Participants were video recorded using the frontal tablet’s camera while tasting the samples to obtain their biometric responses through face recognition and biometric computer vision algorithms. The questionnaire consisted of affective questions using a 15-cm non-structured scale and check all that apply (CATA) using emojis, as shown in Table 1. The use of emojis in the CATA method is a recent improvement in sensory science applied by researchers within the last seven years and has shown to be effective in acquiring conscious responses from consumers^41–47^. Emojis were selected based on previous studies with different food, beverages, and packaging, which have been successful at obtaining emotion data from consumers^10,46–52^.

**Table 1:** Descriptors included in the sensory questionnaire, including the labels used in results and anchors or options used in the scales

Descriptor/question	Label	Anchors/options
Aroma	Aroma	Dislike extremely – like extremely
Texture	Texture	
Bitterness	Bitter	
Sweetness	Sweet	
Aftertaste	Aftertaste	
Face scale*	FaceScale^18^	
Overall liking	Overall	Dislike extremely – like extremely
Check all that apply (Emojis*)	CATA	

^*^Emojis shown for FaceScale were created by the authors. Emojis shown for the CATA question were obtained from the open-source Noto Emoji Font from Google for publication purposes; however, the emojis displayed in the test were the Samsung emojis in color since the BioSensory App uses the emoji Unicodes.

### Biometrics analysis

Videos recorded from participants while tasting samples were analyzed to obtain the subconscious emotional responses using software developed by the Digital Agriculture Food and Wine group from UoM (DAFW-UoM) based on the Affectiva software development kit (SDK; Affectiva, Boston, MA, USA). This software is able to assess head movements and associate facial expressions with emotions and dimensions or states (Table 2).

**Table 2:** Biometrics and descriptors measured from videos and labels used in the paper

Biometric	Descriptor	Label/Image
Head Movements*	Pitch	
	Yaw	
	Roll	
Emotions	Joy	Joy
	Fear	Fear
	Disgust	Disgust
	Sadness	Sadness
	Anger	Anger
	Surprise	Surprise
	Contempt	Contempt
Dimensions/ States	Valence	Valence
	Engagement	Engagement
	Relaxed	Relaxed
Facial Expression	Smile	Smile
Physiological	Heart Rate	HR
	Systolic Pressure	Sys
	Diastolic Pressure	Dias

^*^Images shown for head movements were created by the authors.

Videos were also analyzed for heart rate and blood pressure (systolic and diastolic; Table 2) to obtain physiological responses from participants. These were obtained using a customized algorithm and machine learning models developed by the DAFW-UoM^53^, which is able to measure the luminosity changes in the forehead and cheeks given by the blood flow in these areas.

### Statistical data analysis

Data from the quantitative self-reported and biometric responses were analyzed using one-way ANOVA and Fisher’s least significant difference test (LSD; α = 0.05) to assess significant differences in level two interactions between treatments (seating-position × environment) and treatments and samples (sample × seating-position; sample × environment). Furthermore, the Cochran Q test was conducted for CATA to assess significant differences (*p* < 0.05) for interactions of sample × environment × seating position; Sheskin pairwise comparison test was conducted to assess specific differences between the different samples/treatments. and correspondence analyses (CA) were conducted for CATA to assess the associations between samples/treatments and emojis. Principal components analyses (PCA) were conducted to evaluate relationships between variables and their associations with samples/treatments. The ANOVAs and CAs were conducted using XLSTAT version 2022.3.2 (Addinsoft, New York, NY, USA), while PCAs were developed using Matlab® R2021a (Mathworks, Inc., Natick, MA, USA).

## Supplementary information

Supplementary Material

## Data Availability

Data and intellectual property belong to The University of Melbourne; any sharing needs to be evaluated and approved by the University.
