# Supplementary material for: Exploring consumer acceptability of leafy greens in earth and space immersive environments using biometrics
Source: NPJ Sci Food. 2024 Oct 9;8:81. doi: 10.1038/s41538-024-00314-6 (PMC11464502; doi:10.1038/s41538-024-00314-6)
Supplement: Supplementary file 1 — Supplementary Material [file 41538_2024_314_MOESM1_ESM.pdf]

Table S1. Means and standard errors of the self-reported responses for the interaction of environment and seating position. p-value was obtained from ANOVA test at $p<0.05$.

Interaction	Aroma	Texture	Bitter	Sweet	Aftertaste	FaceScale	Overall
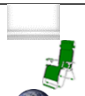	8.60	9.63	8.37	8.76	8.54	9.07	8.90
	±0.44	±0.49	±0.56	±0.54	±0.60	±0.58	±0.57
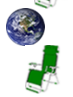	8.90	9.55	8.26	8.58	8.40	8.94	8.86
	±0.45	±0.52	±0.56	±0.54	±0.59	±0.56	±0.57
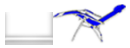	8.84	9.37	8.26	8.80	8.29	8.95	8.68
	±0.43	±0.52	±0.55	±0.54	±0.58	±0.54	±0.55
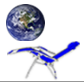	8.93	9.44	8.13	8.55	8.26	8.81	8.74
	±0.45	±0.50	±0.55	±0.56	±0.59	±0.56	±0.59
**p-value**	0.550	0.716	0.969	0.876	0.819	0.985	0.838

Table S2. Means and standard errors of the biometric responses for the interaction of environment and seating position. Abbreviations: HR: heart rate; Sys: systolic pressure; Dias: diastolic pressure; Jaw 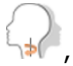 , Pitch 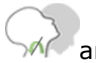 and Roll 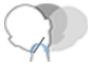 head movements.

Interaction	HR	Sys	Dias	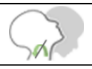	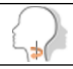	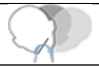	Joy	Fear	Disgust	Sadness	Anger	Surprise	Contempt	Valence	Engagement	Smile	Relaxed
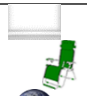	95.31	125.70	76.51	-15.74	3.31	2.18	2.10	0.76	1.39	0.30	0.29	1.36	1.08	-0.89	11.48	3.25	1.62
	±3.40	±6.73	±2.30	±1.09	±0.85	±0.58	±1.11	±0.42	±0.65	±0.16	±0.25	±0.41	±0.58	±1.65	±1.92	±1.36	±0.87
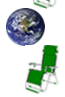	97.48	119.84	75.60	-13.21	4.12	2.74	2.07	0.84	1.32	0.33	0.38	2.45	1.16	-2.18	13.89	2.78	2.14
	±3.67	±8.05	±2.69	±1.04	±0.85	±0.73	±1.17	±0.49	±0.37	±0.22	±0.2	±0.66	±0.45	±1.52	±2.23	±1.30	±1.26
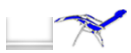	102.39	125.54	76.00	-13.75	2.23	1.66	1.09	0.99	1.20	0.24	0.45	1.79	0.86	-2.55	11.86	1.80	0.79
	±3.42	±7.11	±2.37	±1.22	±0.79	±0.66	±0.93	±0.55	±0.38	±0.17	±0.43	±0.52	±0.3	±1.34	±1.84	±1.01	±0.87
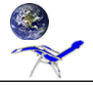	98.53	121.04	75.88	-14.67	1.53	-1.30	0.90	1.87	1.88	0.10	0.24	2.33	0.88	-2.01	11.60	1.62	0.57
	±3.33	±6.85	±2.42	±1.10	±0.80	±0.71	±0.59	±0.6	±0.87	±0.05	±0.30	±0.53	±0.70	±1.28	±1.89	±0.75	±0.41
**p-value**	0.110	0.861	0.763	<0.001	0.045	<0.001	0.851	0.096	0.177	0.238	0.298	0.253	0.913	0.166	0.135	0.781	0.350

Table S3. Means and standard errors of the self-reported responses for the interaction of samples and environment. p-value was obtained from ANOVA test at $p<0.05$.

Interaction	Aroma	Texture	Bitter	Sweet	Aftertaste	FaceScale	Overall
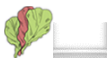	8.44	10.93	9.50	9.96	9.54	10.35	10.09
	±0.34	±0.41	±0.43	±0.42	±0.49	±0.44	±0.44

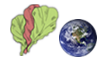	8.71 ±0.34	10.93 ±0.41	9.50 ±0.41	9.96 ±0.39	9.54 ±0.44	10.06 ±0.38	10.09 ±0.40
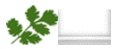	8.49 ±0.51	8.26 ±0.62	8.63 ±0.60	9.07 ±0.58	8.94 ±0.66	9.20 ±0.65	9.12 ±0.65
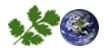	8.73 ±0.48	8.43 ±0.62	8.43 ±0.63	8.62 ±0.61	8.71 ±0.66	8.90 ±0.64	8.84 ±0.66
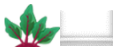	8.21 ±0.25	10.41 ±0.39	8.93 ±0.45	9.34 ±0.45	8.55 ±0.45	9.66 ±0.40	9.25 ±0.43
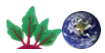	8.44 ±0.29	10.20 ±0.43	8.71 ±0.42	9.23 ±0.45	8.92 ±0.44	9.50 ±0.44	9.43 ±0.44
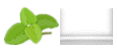	10.22 ±0.53	9.82 ±0.42	7.65 ±0.61	8.44 ±0.57	8.48 ±0.61	8.62 ±0.60	8.64 ±0.60
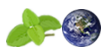	10.19 ±0.49	9.66 ±0.49	8.28 ±0.60	8.58 ±0.61	8.59 ±0.63	9.09 ±0.60	9.08 ±0.60
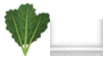	7.90 ±0.31	8.62 ±0.53	7.93 ±0.55	8.17 ±0.53	7.39 ±0.58	8.24 ±0.55	7.74 ±0.55
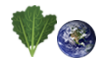	7.93 ±0.37	8.36 ±0.52	7.17 ±0.49	7.39 ±0.46	6.66 ±0.51	7.59 ±0.48	7.19 ±0.51
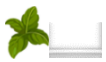	9.04 ±0.56	9.00 ±0.51	7.01 ±0.61	7.72 ±0.63	7.58 ±0.70	7.99 ±0.68	7.81 ±0.65
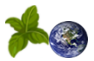	7.90 ±0.54	8.62 ±0.49	7.93 ±0.64	8.17 ±0.63	7.39 ±0.68	8.24 ±0.66	7.74 ±0.67
**p-value**	0.923	0.970	0.697	0.813	0.907	0.837	0.882

Table S4. Means and standard errors of the biometric responses for the interaction of samples and environment. Abbreviations: HR: heart rate; Sys: systolic pressure; Dias: diastolic pressure; Jaw 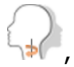 , Pitch 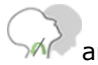 and Roll 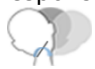 head movements.

Interaction	HR	Sys	Dias	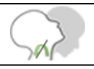	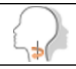	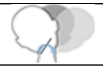	Joy	Fear	Disgust	Sadness	Anger	Surprise	Contempt	Valence	Engagement	Smile	Relaxed
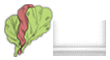	101.88 ±3.57	125.39 ±6.14	76.13 ±2.18	-14.49 ±1.13	3.02 ±0.8	1.86 ±0.62	2.13 ±1.53	0.93 ±0.66	1.03 ±0.22	0.38 ±0.17	0.32 ±0.16	1.62 ±0.58	1.07 ±0.67	-1.03 ±1.72	11.86 ±2.00	2.83 ±1.56	2.28 ±1.55
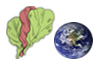	99.24 ±3.33	114.09 ±6.66	73.89 ±2.35	-13.37 ±1.02	3.50 ±1.03	1.13 ±0.89	2.01 ±0.98	1.66 ±0.75	1.14 ±0.25	0.24 ±0.11	0.28 ±0.19	1.78 ±0.5	1.32 ±0.91	-1.13 ±1.26	11.30 ±2.01	2.58 ±1.1	1.97 ±1.09
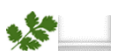	100.21 ±3.14	126.50 ±6.34	77.96 ±2.12	-14.78 ±1.17	2.91 ±0.76	2.15 ±0.57	2.10 ±1.13	1.01 ±0.51	1.67 ±0.97	0.28 ±0.17	0.48 ±0.4	1.80 ±0.58	0.71 ±0.24	-2.28 ±1.9	12.54 ±2.07	3.02 ±1.37	1.42 ±0.93

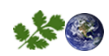	95.01 ±3.37	119.95 ±7.26	74.87 ±2.43	-13.85 ±1.17	2.37 ±0.81	0.43 ±0.73	2.19 ±1.28	1.29 ±0.50	1.68 ±0.70	0.12 ±0.06	0.15 ±0.08	2.73 ±0.7	0.66 ±0.38	-1.68 ±1.7	12.09 ±2.08	2.74 ±1.36	1.60 ±1.11
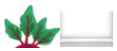	95.44 ±3.06	113.56 ±7.08	73.99 ±2.36	-14.78 ±1.2	2.60 ±0.86	1.62 ±0.62	1.33 ±0.65	0.73 ±0.36	1.38 ±0.32	0.16 ±0.06	0.72 ±0.69	1.55 ±0.32	1.12 ±0.46	-1.62 ±1.39	12.92 ±1.90	2.44 ±1.04	1.28 ±0.76
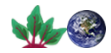	97.10 ±3.68	128.20 ±8.13	76.88 ±2.67	-14.08 ±1.06	2.70 ±0.78	1.20 ±0.81	0.73 ±0.47	1.05 ±0.39	1.19 ±0.27	0.15 ±0.05	0.45 ±0.53	2.48 ±0.69	1.13 ±0.87	-2.44 ±1.22	11.77 ±1.95	1.79 ±0.85	0.62 ±0.36
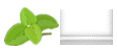	94.01 ±3.35	129.83 ±8.11	76.25 ±2.61	-14.68 ±1.15	3.06 ±0.97	1.84 ±0.69	1.83 ±1.03	0.83 ±0.45	1.19 ±0.38	0.27 ±0.18	0.20 ±0.14	1.09 ±0.27	0.88 ±0.32	-1.13 ±1.44	10.64 ±1.67	2.96 ±1.22	0.85 ±0.45
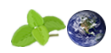	99.30 ±3.38	117.96 ±6.56	75.48 ±2.15	-13.70 ±1.12	3.33 ±0.85	0.50 ±0.70	1.41 ±0.57	1.22 ±0.57	1.09 ±0.19	0.15 ±0.06	0.30 ±0.13	2.63 ±0.56	0.93 ±0.37	-1.60 ±1.13	12.98 ±1.92	2.41 ±0.86	1.54 ±0.73
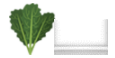	101.14 ±3.71	127.03 ±7.07	75.52 ±2.46	-15.41 ±1.11	2.09 ±0.69	1.48 ±0.62	0.99 ±0.91	0.91 ±0.5	0.91 ±0.18	0.28 ±0.13	0.18 ±0.09	1.30 ±0.29	1.46 ±0.62	-2.37 ±1.29	9.67 ±1.58	1.78 ±0.99	0.55 ±0.55
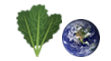	102.21 ±3.87	126.81 ±7.89	78.20 ±2.88	-14.35 ±1.05	1.98 ±0.69	0.84 ±0.74	1.70 ±1.19	1.50 ±0.6	1.82 ±0.56	0.51 ±0.32	0.45 ±0.24	2.39 ±0.46	0.98 ±0.33	-3.45 ±1.5	15.21 ±2.25	2.09 ±1.2	1.54 ±1.17
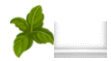	100.40 ±3.73	131.41 ±6.58	77.68 ±2.3	-14.31 ±1.28	2.90 ±0.85	2.59 ±0.63	1.19 ±0.59	0.87 ±0.43	1.60 ±0.64	0.27 ±0.22	0.32 ±0.29	2.09 ±0.63	0.58 ±0.23	-1.88 ±1.14	12.39 ±2	2.12 ±0.86	0.84 ±0.45
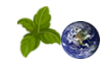	95.16 ±3.16	115.62 ±7.59	75.11 ±2.65	-14.30 ±1.08	3.05 ±0.82	0.20 ±0.74	0.89 ±0.39	1.42 ±0.51	2.68 ±1.43	0.14 ±0.07	0.21 ±0.12	2.33 ±0.56	1.10 ±0.52	-2.29 ±1.43	13.10 ±2.05	1.60 ±0.55	0.86 ±0.44
**p-value**	0.535	0.230	0.635	0.990	0.980	0.393	0.698	0.993	0.664	0.583	0.841	0.534	0.905	0.985	0.248	0.996	0.848

Table S5. Means and standard errors of the self-reported responses for the interaction of samples and seating position. p-value was obtained from ANOVA test at $p<0.05$.

Interaction	Aroma	Texture	Bitter	Sweet	Aftertaste	FaceScale	Overall
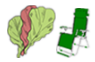	8.70 ±0.30	10.84 ±0.43	9.33 ±0.43	9.80 ±0.43	9.56 ±0.46	10.08 ±0.41	10.03 ±0.41
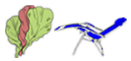	8.45 ±0.37	10.98 ±0.40	9.93 ±0.41	10.11 ±0.39	9.54 ±0.47	10.33 ±0.41	10.24 ±0.43
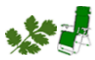	8.56 ±0.49	8.49 ±0.62	8.76 ±0.62	8.81 ±0.60	8.80 ±0.66	9.23 ±0.65	9.12 ±0.65
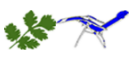	8.67 ±0.50	8.20 ±0.62	8.30 ±0.61	8.88 ±0.60	8.85 ±0.66	8.87 ±0.63	8.84 ±0.65
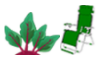	8.26 ±0.24	10.42 ±0.37	8.84 ±0.40	9.22 ±0.41	8.82 ±0.41	9.63 ±0.39	9.38 ±0.39

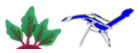	8.40 ±.31	10.19 ±0.44	8.80 ±0.47	9.35 ±0.48	8.65 ±0.48	9.53 ±0.45	9.29 ±0.47
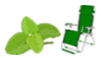	10.15 ±0.53	9.84 ±0.47	8.09 ±0.61	8.62 ±0.60	8.51 ±0.65	8.95 ±0.60	9.03 ±0.60
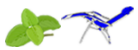	10.26 ±0.49	9.65 ±0.45	7.84 ±0.60	8.40 ±0.58	8.55 ±0.59	8.76 ±0.59	8.70 ±0.60
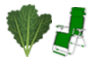	7.69 ±0.33	8.79 ±0.53	7.66 ±0.55	7.91 ±0.52	7.27 ±0.55	8.01 ±0.53	7.60 ±0.55
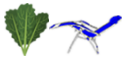	8.13 ±0.35	8.20 ±0.52	7.43 ±0.49	7.65 ±0.48	6.78 ±0.54	7.82 ±0.50	7.33 ±0.52
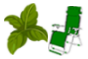	9.14 ±0.54	9.19 ±0.52	7.21 ±0.62	7.68 ±0.61	7.85 ±0.69	8.14 ±0.67	8.13 ±0.65
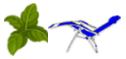	9.37 ±0.56	9.21 ±0.48	6.88 ±0.63	7.67 ±0.65	7.27 ±0.68	7.97 ±0.67	7.84 ±0.68
**p-value**	<0.001	<0.001	<0.001	<0.001	<0.001	<0.001	<0.001

Table S6. Means and standard errors of the biometric responses for the interaction of samples and seating position. Abbreviations: HR: heart rate; Sys: systolic pressure; Dias: diastolic pressure; Jaw 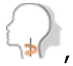 , Pitch 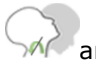 and Roll 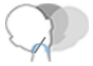 head movements.

Interaction	HR	Sys	Dias	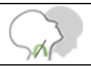	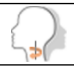	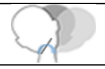	Joy	Fear	Disgust	Sadness	Anger	Surprise	Contempt	Valence	Engagement	Smile	Relaxed
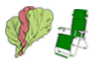	98.45	120.57	76.00	-13.93	4.38	3.14	2.06	0.98	1.05	0.49	0.47	2.08	1.31	-1.95	12.74	2.73	2.28
	±3.82	±6.36	±2.28	±1.05	±0.86	±0.65	±0.94	±0.64	±0.23	±0.2	±0.24	±0.70	±0.74	±1.21	±1.97	±1.07	±1.12
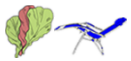	102.67	118.91	74.02	-13.94	2.14	-0.15	2.08	1.61	1.11	0.14	0.13	1.32	1.08	-0.21	10.42	2.68	1.98
	±3.08	±6.48	±2.24	±1.11	±0.94	±0.78	±1.55	±0.75	±0.23	±0.08	±0.08	±0.38	±0.82	±1.75	±2.02	±1.58	±1.53
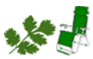	97.11	117.89	75.49	-14.56	3.50	2.71	3.29	0.93	1.79	0.16	0.48	1.76	0.38	-0.64	12.50	4.14	2.91
	±3.32	±7.19	±2.36	±1.19	±0.8	±0.61	±1.56	±0.47	±1.06	±0.08	±0.42	±0.55	±0.09	±2.26	±2.40	±1.80	±1.47
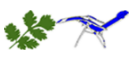	98.11	128.56	77.34	-14.08	1.79	-0.14	1.00	1.36	1.56	0.23	0.15	2.78	0.99	-3.31	12.13	1.62	0.11
	±3.20	±6.3	±2.18	±1.16	±0.75	±0.66	±0.75	±0.54	±0.63	±0.17	±0.13	±0.69	±0.41	±1.3	±1.76	±0.82	±0.07
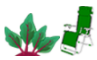	93.61	130.49	77.89	-14.94	3.49	2.27	0.97	0.39	1.39	0.19	0.17	2.15	1.21	-2.52	12.37	2.23	1.39
	±3.51	±8.08	±2.62	±1.01	±0.87	±0.65	±0.58	±0.15	±0.35	±0.06	±0.08	±0.6	±0.5	±1.51	±1.96	±1.09	±0.87
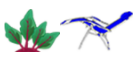	98.93	111.28	72.98	-13.92	1.82	0.55	1.08	1.39	1.18	0.11	1.00	1.89	1.04	-1.53	12.32	1.99	0.50
	±3.13	±6.86	±2.34	±1.23	±0.77	±0.75	±0.58	±0.48	±0.25	±0.05	±0.83	±0.45	±0.79	±1.15	±1.90	±0.85	±0.29
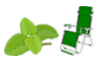	96.40	124.67	75.81	-13.79	4.44	2.23	2.54	0.77	1.29	0.32	0.37	1.70	1.23	-0.93	13.72	3.76	1.72
	±3.27	±8.17	±2.59	±1.14	±1.01	±0.65	±1.2	±0.41	±0.35	±0.19	±0.18	±0.43	±0.44	±1.55	±2.08	±1.44	±0.73

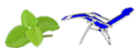	96.92	123.12	75.92	-14.59	1.95	0.12	0.69	1.28	0.99	0.09	0.13	2.01	0.58	-1.79	9.90	1.60	0.68
	±3.5	±6.78	±2.23	±1.13	±0.8	±0.72	±0.33	±0.58	±0.27	±0.06	±0.08	±0.45	±0.21	±1.06	±1.46	±0.57	±0.43
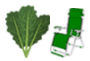	97.70	118.52	74.78	-15.13	3.10	2.13	2.16	0.86	1.21	0.60	0.40	1.87	1.47	-1.88	12.72	2.93	1.74
	±3.69	±7.34	±2.63	±0.98	±0.69	±0.62	±1.48	±0.47	±0.46	±0.33	±0.21	±0.4	±0.66	±1.71	±2.09	±1.53	±1.26
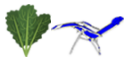	105.66	135.33	78.95	-14.63	0.97	0.19	0.53	1.54	1.52	0.19	0.24	1.82	0.97	-3.94	12.17	0.93	0.35
	±3.80	±7.43	±2.69	±1.16	±0.66	±0.70	±0.38	±0.61	±0.35	±0.09	±0.14	±0.36	±0.34	±1.02	±1.81	±0.43	±0.32
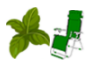	95.10	124.48	76.36	-14.49	3.37	2.27	1.49	0.87	1.41	0.13	0.12	1.87	1.10	-1.28	12.07	2.27	1.24
	±3.47	±6.68	±2.37	±1.15	±0.85	±0.69	±0.67	±0.42	±0.43	±0.08	±0.06	±0.47	±0.53	±1.09	±1.85	±0.86	±0.56
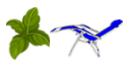	100.46	122.54	76.42	-14.12	2.59	0.51	0.60	1.42	2.87	0.28	0.41	2.55	0.58	-2.89	13.42	1.44	0.46
	±3.47	±7.59	±2.58	±1.22	±0.83	±0.7	±0.33	±0.5	±1.36	±0.22	±0.31	±0.68	±0.22	±1.39	±2.15	±0.63	±0.33
**p-value**	0.862	0.119	0.447	0.942	0.818	0.580	0.490	0.989	0.437	0.126	0.122	0.284	0.704	0.307	0.612	0.599	0.553

Table S7. Results from Cochran Q test including p-value (criteria: $p < 0.05$) for the interaction of sample × environment × seating position. Letters a-c depict significant differences based on pairwise comparison test using Sheskin test, those with no letters were identified as similar (letter a for all) by the Sheskin test.

Interaction	😀	🤔	🙂	😱	😍	😄	🤒	😆	😐	😵	😋	😉	😌	🤗	😝	🙄	😴	😮
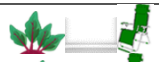	0.14	0.31	0.33	0.00[a]	0.02	0.08	0.02	0.26	0.29[ab]	0.00[a]	0.16	0.04	0.28	0.06	0.10[ab]	0.06	0.00	0.00
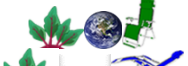	0.18	0.28	0.24	0.00[a]	0.02	0.16	0.02	0.14	0.33[ab]	0.02[ab]	0.08	0.02	0.24	0.08	0.02[a]	0.10	0.00	0.02
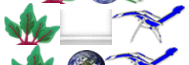	0.20	0.12	0.37	0.00[a]	0.04	0.20	0.00	0.22	0.29[ab]	0.02[ab]	0.14	0.08	0.14	0.08	0.04[ab]	0.08	0.02	0.00
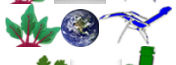	0.12	0.14	0.24	0.06[ab]	0.02	0.20	0.02	0.33	0.26[ab]	0.08[abc]	0.20	0.04	0.24	0.10	0.04[ab]	0.08	0.00	0.02
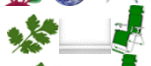	0.28	0.12	0.26	0.08[ab]	0.16	0.20	0.02	0.24	0.10[a]	0.16[abc]	0.28	0.06	0.33	0.12	0.12[ab]	0.10	0.00	0.04
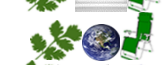	0.18	0.18	0.29	0.12[ab]	0.10	0.18	0.08	0.24	0.16[ab]	0.16[abc]	0.14	0.12	0.16	0.14	0.14[ab]	0.16	0.00	0.06
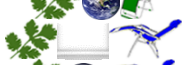	0.20	0.12	0.24	0.10[ab]	0.06	0.16	0.04	0.33	0.18[ab]	0.14[abc]	0.26	0.10	0.18	0.12	0.12[ab]	0.14	0.00	0.06
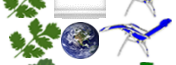	0.26	0.24	0.16	0.10[ab]	0.14	0.18	0.02	0.29	0.16[ab]	0.18[abc]	0.20	0.04	0.16	0.18	0.14[ab]	0.10	0.02	0.02
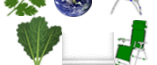	0.20	0.18	0.22	0.10[ab]	0.04	0.10	0.04	0.18	0.22[ab]	0.20[abc]	0.10	0.04	0.18	0.02	0.16[ab]	0.06	0.00	0.06
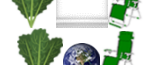	0.10	0.24	0.24	0.10[ab]	0.04	0.04	0.04	0.10	0.39[ab]	0.16[abc]	0.08	0.02	0.18	0.04	0.22[ab]	0.08	0.00	0.04
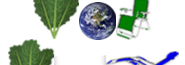	0.10	0.20	0.14	0.08[ab]	0.08	0.08	0.06	0.22	0.45[b]	0.20[abc]	0.06	0.02	0.12	0.06	0.20[ab]	0.02	0.00	0.02
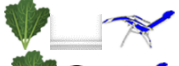	0.04	0.20	0.28	0.10[ab]	0.04	0.04	0.08	0.10	0.29[ab]	0.20[abc]	0.10	0.00	0.20	0.00	0.24[b]	0.12	0.00	0.08
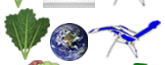	0.24	0.14	0.33	0.02[ab]	0.08	0.22	0.00	0.31	0.16[ab]	0.08[abc]	0.20	0.14	0.20	0.08	0.02[a]	0.16	0.02	0.04
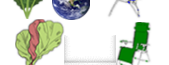	0.16	0.14	0.31	0.02[ab]	0.02	0.10	0.04	0.33	0.26[ab]	0.06[abc]	0.22	0.10	0.20	0.14	0.04[ab]	0.10	0.00	0.00
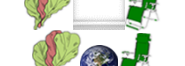	0.24	0.14	0.16	0.00[a]	0.14	0.28	0.02	0.37	0.18[ab]	0.04[abc]	0.10	0.10	0.20	0.12	0.02[a]	0.10	0.02	0.00
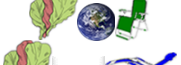	0.20	0.14	0.29	0.00[a]	0.12	0.20	0.06	0.35	0.22[ab]	0.04[abc]	0.24	0.10	0.22	0.14	0.02[a]	0.10	0.00	0.02

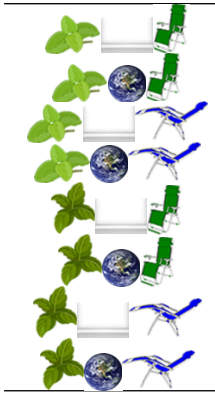	0.20	0.16	0.29	0.10^{ab}	0.10	0.10	0.08	0.28	0.26^{ab}	0.08^{abc}	0.20	0.04	0.22	0.10	0.04^{ab}	0.12	0.00	0.04
	0.18	0.20	0.20	0.06^{ab}	0.12	0.12	0.02	0.33	0.24^{ab}	0.14^{abc}	0.22	0.04	0.16	0.08	0.08^{ab}	0.12	0.02	0.00
	0.14	0.26	0.10	0.12^{ab}	0.16	0.14	0.08	0.31	0.31^{ab}	0.12^{abc}	0.20	0.04	0.08	0.06	0.10^{ab}	0.06	0.04	0.00
	0.16	0.16	0.22	0.06^{ab}	0.14	0.18	0.04	0.33	0.18^{ab}	0.20^{abc}	0.26	0.10	0.22	0.06	0.06^{ab}	0.14	0.00	0.02
	0.20	0.18	0.24	0.08^{ab}	0.10	0.18	0.04	0.20	0.12^{a}	0.26^{c}	0.16	0.10	0.14	0.08	0.16^{ab}	0.16	0.02	0.02
	0.18	0.14	0.16	0.14^{ab}	0.14	0.16	0.06	0.31	0.14^{a}	0.16^{abc}	0.16	0.08	0.18	0.08	0.20^{ab}	0.14	0.00	0.06
	0.20	0.22	0.14	0.20^{b}	0.16	0.14	0.06	0.28	0.16^{ab}	0.20^{abc}	0.12	0.06	0.14	0.06	0.16^{ab}	0.08	0.00	0.04
	0.18	0.06	0.24	0.20^{b}	0.14	0.16	0.10	0.29	0.12^{a}	0.24^{bc}	0.20	0.06	0.16	0.08	0.14^{ab}	0.08	0.00	0.06
**p-value**	0.112	0.118	**0.023**	**<0.001**	**0.001**	**0.035**	0.392	**0.005**	**<0.001**	**<0.001**	**0.004**	0.201	0.255	0.167	**<0.001**	0.664	0.445	0.349
